# A Medical Duel

**Published:** 1894-12-22

**Authors:** 


### A Medical Duel.

Whether Richer, the monk, used his skill in the monastic infirmary or hospital we do not know; but he loses no opportunity of referring to medical subjects in his "History of His Own Times." A striking example is the following, which is further interesting as being the earliest notice of a Salernitan physician, while it incidentally throws some light on the vexed question of the origin of that famous medical school. This, so far as it goes, supports the modern view that the school was a lay institution from the first, for the Salernitan is contrasted with his clerical opponent as being a good practical man indeed, but illiterate, and without knowledge of the learned languages.

"In A.D. 946 died Derold, Bishop of Amiens, an honourable courtier beloved of the king, and very skilled in medicine. The story is told that, when he served the king, he had a contest in cunning with a certain Salernitan. Both were excellent physicians, but while Derold was in greater favour with the king, the queen considered the Salernitan more skilful. So the king contrived a way to test their respective ability without their knowing it. He invited both to dinner, and put questions to them on medical matters. They answered as best they could; Derold being a man of learning gave satisfactory definitions, while the Salernitan, though he had no literary knowledge, posssessed great talent and practical experience. By royal command they came every day to dinner, and sat next to one another. Now they were disputing one day over the potencies (dynamidia) of drugs, and discussed the respective provinces of pharmaceutics, botanics, and chirurgics. The Salernitan had never heard those strange names, so he blushed and was silenced. Henceforth he became so jealous that he plotted to poison his rival, while pretending special friendship for him. Having prepared a poison, he anointed the nail of his middle finger therewith, and put it in the pepper water (liquorem piperis) in which they both dipped their food. Derold carelessly tasting therefrom, soon began to feel unwell, so he was led out by his friends, and cured himself in three days with theriac. On his return, he concealed his knowledge of the trick, and when the Salernitan inquired after his health, said he had had a slight catarrh, treating him with great courtesy so as to put him off his guard. Then he (Derold) strewed some poison, which he held between his little and ring fingers, on his neighbour's meat, which being absorbed drove out the vital heat, and the sick Salernitan was led away by his friends. He tried to expel the poison, but in vain. Now he praises Derold as the best of physicians, and entreats his aid. Derold, at the king's command, gave him antidotes, but purposely left the cure incomplete, for the theriac drove the poison down into his left foot, where it caused a swelling, and afterwards an open wound, so that, finally, his foot was miserably amputated by the surgeons."

Richer devotes great care to the exact description of the last illnesses of his more celebrated contemporaries, and in some cases makes an obvious attempt to imitate the clinical histories of Hippocrates. The following account of the death of the Emperor Otto II. shows that the huge doses of purgatives employed by our mediæval ancestors were not always without serious effects.

In 983 Otto, being at Rome, suffered from an indigestion, with scybala (squibalas) of the intestine, due to excess of black bile. Eager to cure himself he took four drachms of aloes in a single dose. Hence he got severe colic, continued diarrhœa, and hæmorrhoids. Excessive loss of blood through these caused his death in a few days, December 7th.

In the following, which is an interesting case for diagnosis, the abrupt Hippocratic style is very marked.

In the spring of the year 986 King Lothaire fell sick at Laon, owing, as Richer thinks, to a sudden change in the weather, the winter having been remarkably mild, followed by a cold spring. "He was attacked by the disease physicians call colic. He suffered extreme pain in the right iliac region, together with similar intermittent pains spreading from the umbilicus towards the spleen, and thence downwards to the left groin and fundament. Pain in the region of the kidneys, continued straining and tenesmus, bloodstained motions, intermittent rigors, loud rumbling noises in the abdomen, perpetual nausea with vain attempts at vomiting; finally swelling of the abdomen with ardor stomachi. The whole palace was filled with groans and lamentations. None who saw him could refrain from weeping. So died Lothaire on March 11th."

His son, the last monarch of the house of Charlemagne, soon followed him. "The king was amusing himself with hunting near Senlis, when his foot slipped and he fell, causing himself great pains in the liver. Now, since the liver, as physicians say, is the seat of the blood, its concussion resulted in a vomiting of blood in large quantity both from nose and mouth. Then he got severe pains and palpitations in the chest and great heat of the whole body. So he died and paid the debt of nature May 22nd, having survived his father one year."

The worthy monk takes especial delight in recording the miserable ends of the wicked, especially those who had done violence to the clergy. Some of these accounts are too horrible for translation, but the following is his story of the death of Heribert, a French noble "who feared not God neither regarded man," or, in other words, refused to obey either bishop or king: "While he was preparing for further wickedness, seated in the midst of his followers, in glorious apparel, and addressing them with extended arm, he was seized with the greater apoplexy, due to a superfluity of humours. His fingers became clenched, his muscles contracted, his mouth was drawn towards one ear, and so he fell dead without warning before all his men, whose hair stood on end with horror."

### HIGH-PRESSURE BOILERS.

The Board of Trade has no control over the use of high-pressure boilers in private houses, and until it is provided with necessary powers we shall continue to hear of explosions and loss of life after or during frosts in the winter months. The use of safety-valves should be compulsory, and the placing of printed instructions in every kitchen where high-pressure boilers are in use might do still more.

